# Genome Sequence and Annotation of Helicobacter pylori Strain Hp238, Isolated from a Taiwanese Patient with Mucosa-Associated Lymphoid Tissue Lymphoma

**DOI:** 10.1128/genomeA.00006-15

**Published:** 2015-02-19

**Authors:** Cheng-Yen Kao, Jenn-Wei Chen, Yi-Ting Huang, Shew-Meei Sheu, Bor-Shyang Sheu, Jiunn-Jong Wu

**Affiliations:** a Department of Medical Laboratory Science and Biotechnology, National Cheng Kung University, Tainan, Taiwan; b Center of Infectious Disease and Signaling Research, National Cheng Kung University, Tainan, Taiwan; c Department of Internal Medicine, College of Medicine, National Cheng Kung University, Tainan, Taiwan

## Abstract

We present the complete genome sequence of Helicobacter pylori strain Hp238, isolated from a Taiwanese patient with gastric mucosa-associated lymphoid tissue lymphoma. Importantly, H. pylori strain Hp238 can multiply in THP-1 cells after internalization through the induction of autophagosome formation. These genome data will help to identify genes associated with H. pylori intracellular multiplication and pathogenesis.

## GENOME ANNOUNCEMENT

Helicobacter pylori is a Gram-negative spiral-shaped microaerophilic bacterium that infects 50% of the population worldwide (1). Persistent infection with H. pylori increases the risk of developing gastroduodenal diseases, including peptic ulcer, duodenal ulcer, and gastric adenocarcinoma (2, 3). The most common histopathological features of gastric malignancies are adenocarcinoma and lymphoma of mucosa-associated lymphoid tissue (MALT). It has been calculated that the risk of gastric adenocarcinoma and MALT lymphoma in H. pylori-infected individuals is 3- to 6-fold higher than in those who are uninfected (4). Genome diversity, especially involving disease-associated genes, is highly associated with different gastroduodenal diseases, since H. pylori has coevolved with human populations (5). The study of whole-genome sequences from strains isolated from patients with MALT lymphoma is necessary to better understand the pathogenesis of the disease and to identify virulence genes useful in the identification of patients with higher risk.

We report the genome sequence of H. pylori strain Hp238, isolated from a 57-year-old Taiwanese man diagnosed with MALT lymphoma. In a previous study, we determined that Hp238 can multiply in THP-1 cells after internalization through the induction of autophagosomes formation (6). Although CagA and VacA participate in Hp238 multiplication in THP-1 cells, whether other virulence factors are involved in this multiplication remained unclear. This genome sequence will provide valuable information for a better understanding of bacterial pathogenesis. Particularly in Taiwan, this is the first report of a genome sequence of H. pylori.

The Hp238 strain was sequenced using the 454 junior platform (Roche, Germany), generating a library containing 252,098 single reads with an average length of 405 bp and a 33-fold average coverage. The genome sequence was generated by a *de novo* assembly using the GS Assembler version 2.6 software (Roche). This strategy provided 80 large contigs. As a reference, we employed the genome sequence of H. pylori strain B38 (NC_012973.1). The contigs were then remapped according to a previous study (7), and the results showed two scaffolds with 67 gaps. Finally, gaps were filled in through PCR and direct sequencing. The Hp238 genome has a size of 1,586,473 bp and a GC content of 38.7%.

Sequences were annotated at NCBI (http://www.ncbi.nlm.nih.gov/genome/annotation_prok; strain Hp238) or with the RAST prokaryotic genome annotation server (http://www.nmpdr.org/FIG/wiki/view.cgi/Main/RAST). RAST annotation results showed that the genome contained 92.3% coding regions and 1,663 genes, including 39 RNA genes. The average length for protein-coding genes was found to be 897 bp. The results showed the presence of major virulence markers, including *cagA* (typing of the repeating EPIYA motifs revealed the ABD type), *vacA* (typing of s and m regions, s1c/m2), and *babA*. Moreover, genome and gene comparison analysis by RAST with other fully and partially sequenced strains demonstrates that this genome is most similar to the H. pylori 51 strain isolated in eastern Asia (Korea), followed by H. pylori strains Shi470 and 35A.

### Nucleotide sequence accession number.

The complete genome sequence of strain Hp238 has been deposited in GenBank under the accession number CP010013.
